# Modelling collective cell migration: neural crest as a model paradigm

**DOI:** 10.1007/s00285-019-01436-2

**Published:** 2019-10-05

**Authors:** Rasa Giniūnaitė, Ruth E. Baker, Paul M. Kulesa, Philip K. Maini

**Affiliations:** 1 Wolfson Centre for Mathematical Biology, Mathematical Institute, University of Oxford, Woodstock Road, Oxford, OX2 6GG UK; 2 Stowers Institute for Medical Research, 1000 E 50th Street, Kansas City, MO 64110 USA

**Keywords:** Collective cell migration, Domain growth, Individual-based models, Partial differential equations, Neural crest, 92

## Abstract

A huge variety of mathematical models have been used to investigate collective cell migration. The aim of this brief review is twofold: to present a number of modelling approaches that incorporate the key factors affecting cell migration, including cell–cell and cell–tissue interactions, as well as domain growth, and to showcase their application to model the migration of neural crest cells. We discuss the complementary strengths of microscale and macroscale models, and identify why it can be important to understand how these modelling approaches are related. We consider neural crest cell migration as a model paradigm to illustrate how the application of different mathematical modelling techniques, combined with experimental results, can provide new biological insights. We conclude by highlighting a number of future challenges for the mathematical modelling of neural crest cell migration.

## Introduction

Migration of cells is crucial in many areas of biology and medicine, including embryonic development, cancer, wound healing and tissue regeneration (Friedl and Wolf 2010). In most of these biological systems, cells migrate in groups as a result of cell–cell communication and by cell–tissue interactions; a phenomenon known as collective cell migration. Understanding the mechanisms that underpin and control collective cell migration can, potentially, enable us to prevent mistargeted or uncontrolled migration, which can result in abnormalities such as developmental defects or cancer metastasis.

One of the key challenges to understanding collective cell migration arises due to the multiscale nature of the phenomenon where, by multiscale, we mean the interaction of processes ranging from intracellular to tissue-level. Collective cell migration is usually characterised not only by cell–cell communication, but also cell–tissue interactions, which require careful understanding of the surrounding tissue through which, or on which, the cells are moving. Interactions can range from local, via cell body contact, to highly non-local, via extended cell protrusions (Tucker and Erickson 1986; Teddy and Kulesa 2004). In addition, gene expression profiles can vary across cells, and it is an open question as to how great a role heterogeneity in cell genotype and phenotype plays in collective migration. All these phenomena combined make collective cell migration highly complex, and individual fields, including biology, bioinformatics and mathematics, have to be integrated to understand the process to the fullest extent.

Recent advances in biotechnology have contributed to an increase in availability of quantitative biological data on cells, such as individual cell trajectories and cell gene expression. To process such large amounts of data on highly complex interacting systems and reach valid conclusions in many cases requires us to move beyond verbal reasoning, and use mathematical models. In the context of collective cell migration, the increasing number of experimental observations over many different scales requires the use of different modelling frameworks, from cell-level individual-based models (IBMs) to high-level partial differential equation (PDE) models [see, for example, the reviews by Danuser et al. (2013) and Markham et al. (2014)]. The integration of different modelling frameworks is essential to bridge the gap between parts of the system evolving on different time and space scales [see the reviews by Banasiak and Miekisz (2008) and Burini and Chouhad (2019)], while comparison of models allows us to evaluate their strengths and weaknesses. Moreover, the ability to translate from cell-level IBMs to PDEs facilitates analyses of the same system from different perspectives and ensures that the continuum model incorporates mechanistic cell properties. In this review, we will provide in a nutshell a summary of the modelling techniques for collective cell migration, and briefly discuss the relations and derivations of different modelling frameworks. It is important to note that we will focus on migratory streams of cells in which the cells are separated from each other, allowing them to move more freely as individuals, for example, as in long distance migration of some metastatic cancer cells (Kedrin et al. 2008). Migratory streams of cells can also be composed of tightly packed cells that move in continuous sheets, for example, cells within epithelial tissue (Freshney and Freshney 2004), but models of this type of migration are beyond of the scope of this review.

**Fig. 1:**
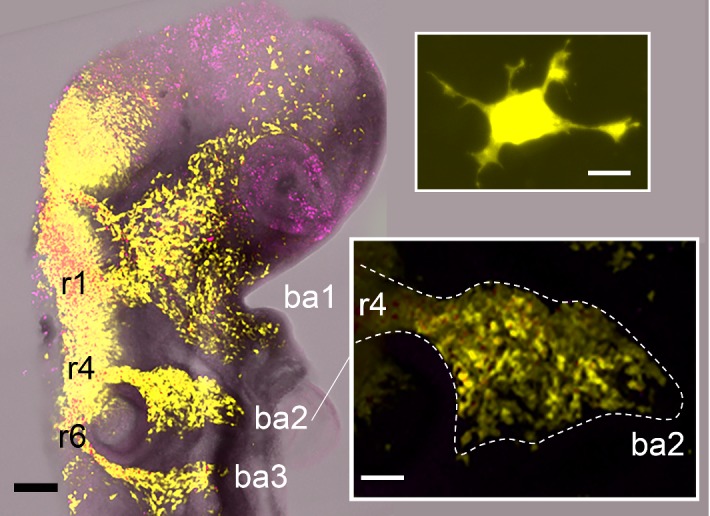
Neural crest cell migratory streams in the chick head co-labelled with pMES (EGFP) in yellow and DiI in purple. Rhombomeric segments of the hindbrain, from which some of the cranial NC cells emerge are labelled rhombomere 1 (r1), r4, and r6 with branchial arch target sites, branchial arch 1 (ba1), ba2, ba3 and the scalebar is 200 microns (black). The top inset is of a typical individual migrating neural crest cell with scalebar of 10 microns (white). The bottom inset is the neural crest cell migratory stream adjacent to r4 and the scalebar is 100 microns (white) (color figure online)

To showcase a wide range of applications of different modelling frameworks for collective cell migration, we choose the embryonic neural crest (NC), a system which encompasses most of the aforementioned challenges that arise in the quest to understand collective cell migration. NC cells are multipotent, highly migratory cells that delaminate from the neural tube all along the vertebrate axis and traverse along well-defined migratory routes to precise targets, where they differentiate (Fig. 1). We choose the NC to exemplify diverse mechanisms that drive collective cell migration because, even within a single species, a wide range of these mechanisms are displayed by distinct types of NC cells that emerge at different locations (for example; cranial, cardiac, vagal, and trunk), and are affected by different signalling pathways [see, for example, the reviews by Theveneau et al. (2010), Schumacher et al. (2016) and Szabó and Mayor (2016, 2018)]. Moreover, there are further differences across different species (Barriga et al. 2015). In addition, collective migration of NC cells has been studied not only to understand embryonic development and the reasons for developmental defects, but also to gain insights into metastatic cancer. This is possible due to the remarkable similarities between the gene expression profiles of migrating NC cells and highly invasive tumour cells (Theveneau and Mayor 2011; Kulesa et al. 2013). Experiments on, and observations of, in vivo cancer cells are highly challenging, so the study of the more experimentally tractable system of NC can provide clues for potential therapeutic intervention in cancer.

The great variety of different mechanisms of migration and experimental tractability of the NC system ensures the generation of extensive biological data, which can be used to derive experimental hypotheses. Due to the fact that the system exhibits complex, nonlinear, local and non-local feedback, it is typically the case that the hypotheses are verified using computational modelling, and, in turn, model predictions can be tested through custom-designed experiments. The similarities of NC cells and metastatic cancer cells demonstrate that interdisciplinary studies of NC not only stimulate new research in mathematical modelling of collective cell migration, but also contribute to a broad range of new biology. Therefore, as well as being of interest in its own right, NC serves as an important paradigm system for collective cell migration.

The remainder of this paper is organised as follows. We begin, in Sect. 2, with a discussion of different types of IBMs that are used to model collective cell migration. In Sect. 3 we discuss how PDE models are used to describe collective cell migration and note that they may, in some cases, be derived from IBMs. In Sect. 4 we introduce integro-PDE models and explain how they have been used to describe collective cell migration. We proceed with some relevant theory on growing domains to account for cases of cell migration within growing tissues in Sect. 5. In Sect. 6 we summarise different computational models used for NC cell migration and provide a motivational example for future research. We conclude with a short discussion and future directions in Sect. 7.

## Individual-based models

The enormous recent advances in biotechnology allow experimentalists to observe individual cell behaviours and quantify some of the key cell specific parameters such as, for example, cell size and shape, and the number of filopodia a cell extends to explore its environment. To incorporate this detail at the cell level, stochastic IBMs are frequently used. In an IBM each cell is treated as a distinct entity with individual properties, such as its ability to interact with other cells and its environment, which can be encoded via a set of rules and/or equations of motion based on these properties [see the book by Anderson et al. (2008); and the reviews in the books by Alber et al. (2003) and Drasdo (2003)]. Cell movement in an IBM can be either restricted to lattice sites, in this case the models are called lattice-based, or the cells can move freely anywhere in space, these models are called off-lattice. Lattice-based models are commonly used because they are conceptually simple and computationally efficient (Simpson et al. 2013). Off-lattice models (Codling et al. 2008; Galle et al. 2005; Newman and Grima 2004) offer more flexibility, but they can be computationally expensive to simulate and difficult to analyse. By way of example, we present two categories of lattice-based models: cellular automaton (CA) and Cellular Potts (CP) models, and a selection of off-lattice models.

*Cellular automaton (CA)* These models were introduced by Neumann and Ulam in the 1950s as a model of individual reproduction (Burks 1970). In CA models, individual cell behaviour and cell–cell interactions are described by rules. The most common processes modelled in this way are proliferation and movement. Two important characteristics of CA models—simplicity and efficient parallel computation—justify the wide use of this framework to model collective cell migration [see the books by Deutsch and Dormann (2005, 2018), Chopard (2012) and the review by Hatzikirou et al. (2012)]. There have been multiple extensions of the simple CA model, such as asynchronous CA (Badoual et al. 2010) and lattice-gas CA (Bussemaker 1996), which enable the model to account for more complex cell–cell and cell–environmental interactions.

*Cellular Potts (CP) models* In the CP model each cell is a subset of lattice sites sharing the same cell identity, i.e. a cell is made up of parts and so a cell can change shape (Graner and Glazier 1992). The algorithm is updated by choosing a random lattice site, proposing a movement and then deciding whether to accept it based on a Hamiltonian function, consisting of a volume constraint term responsible for maintaining an approximately constant cell volume, and a surface energy term responsible for cell–cell adhesion properties. Other terms can be added to the Hamiltonian to account for other interactions. The key advantage of CP models is their ability to resolve cell shape, which accounts for the cell level detail, enabling them to provide a representation of the cellular microenvironment (Szabó and Merks 2013).

*Off-lattice IBMs* The disadvantages experienced by lattice-based models due to lattice effects can be resolved using off-lattice models. In off-lattice models there are a number of ways to represent cells, either as points, spheroids, or more complex, deforming shapes (Woods et al. 2014). Cell position evolves in time due to the action of force laws governing the mechanical interactions between individual cells and cell–tissue interactions, such as volume exclusion, meaning that a cell cannot occupy space that is already occupied by another cell, co-attraction and chemotaxis. The studies of off-lattice models include Newman (2007), Macklin et al. (2012), Yangjin et al. (2007) to mention but a few. This type of modelling framework allows for detailed realistic representations of cells, but there is a trade-off between biological realism and computational cost.

IBMs form a framework that allows for the explicit incorporation of cell-level, biological detail, but at the same time, via cell–cell and cell–tissue interactions, it enables all cells to act as one collective body. This leads to biologically realistic models for collective cell migration. However, the main limitation of IBMs is that they can be less mathematically tractable than continuum models, which we will discuss in the following section.

## Partial differential equation models

PDE models assume that populations can be modelled as continuous entities, and a strength of this approach is the large number of analytic results one can bring to bear on the resultant models. Moreover, they provide a mathematically consistent framework in which the effects of different model hypotheses proposed at the microscopic (cell) level, can be seen and compared at the macroscopic (tissue) level. However, it should be noted that the complexity of the underlying biology can lead to fully nonlinear systems of PDEs for which there are few rigorous results, and many open questions.

Perhaps the most famous PDE in mathematical biology is the diffusion equation, which has a long history of application to model collective cell motility. In this framework, global population migration is assumed to be induced by individuals spreading out as a result of random movements. There are many ways to derive the diffusion equation from random processes (Murray 2002). One method involves the derivation of the telegraph equation from a stochastic velocity-jump process, in which there are discontinuous changes in the speed or direction of a cell, and then taking an appropriate limit (Taylor 1922; Goldstein 1951; McKean 1967; Kac 1974; Segel 1978; Othmer et al. 1988). It is assumed that cells move along the *x*-axis at a constant speed *s* and at random times they reverse direction according to a Poisson process with constant intensity $\lambda$ (Othmer et al. 1988; Othmer and Hillen 2000). It can be shown that the resultant cell density *p*(*x*, *t*) satisfies

1 $$\begin{aligned} \frac{\partial ^2 p}{\partial t^2} + 2 \lambda \frac{\partial p}{\partial t} = s^2 \frac{\partial ^2 p}{\partial x^2}. \end{aligned}$$

Rescaling time and space with $\tau = \epsilon ^2 t$ and $\xi = \epsilon x$ in Eq. (1), respectively, where $\epsilon$ is a small parameter, gives

2 $$\begin{aligned} \epsilon ^2 \frac{\partial ^2 p}{ \partial \tau ^2} + 2\lambda \frac{\partial p}{\partial \tau } = s^2 \frac{\partial ^2 p}{\partial \xi ^2}. \end{aligned}$$

Then, in the limit $\epsilon \rightarrow 0$, i.e. time and space variables tending to zero, we obtain the diffusion equation

3 $$\begin{aligned} \frac{\partial p}{\partial t} = D \frac{\partial ^2 p}{\partial x^2}, \end{aligned}$$

with $s^2/ 2\lambda \equiv D$. This method of rescaling reveals how space and time scales must be related for a diffusive process.

Note that this approach to derive the telegraph equation is only possible in one dimension, but the theory of mixtures can be used to obtain the telegraph equation in any number of dimensions (Othmer 1976). There has been a significant focus on how Eq. (1) is modified when the cell dynamics are also affected by chemotaxis (Stevens and Othmer 1997; Painter et al. 2000; Erban and Othmer 2004, 2007). For example, Erban and Othmer (2004) considered the changes in dynamics when the turning rate is assumed to be a functional of the internal state of the cell. Using moment closure techniques they derived and analysed a system of hyperbolic differential equations, which exhibited results consistent with Monte Carlo simulations of individual movements (Setayeshgar et al. 2005). This example shows how the construction of PDE models from stochastic processes allows for the integration of microscopic intracellular processes, such as signal transduction, into macroscopic parameters, such as chemotactic sensitivity.

Many other different techniques have been developed to derive macroscopic (tissue-level) models from microscopic (cell-level) descriptions (Gavagnin and Yates 2018). A general methodology to derive a PDE description from lattice-based IBMs includes writing down the continuous time occupancy master equation and taking an appropriate limit in lattice spacing and time step. The resulting continuum limit can take many different forms depending on the assumptions included in the IBM. Mean-field and moment dynamics approximations are used to derive continuum limits from stochastic lattice-based and off-lattice models (Markham et al. 2015; Matsiaka et al. 2018).

Continuum limits from lattice-based IBMs have been used to justify the use of PDEs to model diffusion, chemotaxis, exclusion processes, etc. (Baker and Simpson 2010; Binder et al. 2008; Muhuri et al. 2011; Alber et al. 2007; Simpson et al. 2011; Lushnikov et al. 2008; Chauviere et al. 2012). A wide range of nonlinear advection–diffusion equations that, at the macroscopic cell population level, incorporate different assumptions on the dynamics of cells at the individual level, have been derived from lattice-based models by Penington et al. (2011), who provided functional forms of the diffusion term, which incorporates different levels of complexity, from neighbour-based movement to myopic exclusion processes. For the simple single-species cases, in the continuum limit, Penington et al. (2011) obtained the general equation

4 $$\begin{aligned} \frac{\partial p}{\partial t} = D_0 \nabla \cdot (D(p)\nabla p), \end{aligned}$$

where $p(\varvec{x},t)$ is the density of cells at position $\varvec{x} \in {\mathbb {R}} ^d$ at time *t*, $\forall d \in \mathbb {N}$, where *d* is the number of spatial dimensions, and $D_0$ is the constant single-agent diffusivity. They showed that different forms of the diffusivity factor, *D*(*p*), arise depending on the assumptions made for the transition probabilities between lattice sites associated with the discrete (cell-level) model. This approach creates a framework in which we can move away from abstracted phenomenological arguments used to support different proposed forms for diffusion coefficients in continuum models to those derived from detailed biological considerations.

The ability to derive PDEs from cell-level details opens up paths to provide analytical results on the collective invasion of cells. For example, Johnston et al. (2017) derived twenty-two different classes of PDEs from processes with different birth, death and movement rates for isolated individuals and individuals in groups. They examined the ability of each class of PDEs to support travelling wave solutions and considered long time behaviour in terms of individual-level parameters. Johnston et al.’s (2017) main analytical result revealed that a strong Allee effect and nonlinear diffusion can lead to shock-fronted travelling waves, where a shock-fronted travelling wave is defined as a sharp change in the function describing cell density that moves with constant speed. This allows classification of the behaviour of populations with different group and individual motility rates, and provides conditions for successful collective invasion. There are further questions about the sensitivity of the behaviour of these macroscopic nonlinear diffusion models to changes in cell-level details. For example, in Eq. (4) an open question concerns how the nonlinear form of *D*(*p*) affects the ability of the system to exhibit travelling wave solutions, and the properties of these solutions if they exist. Answers to these questions would help to determine how accurately we need to measure and categorise cell-level behaviour in order to include all significant features in continuum models.

Significant progress has also been made in the derivation of continuum PDEs from IBMs for off-lattice models. For example, Dyson et al. (2012) used moment-closure approximations to derive a continuum description of a motile cell population from an off-lattice IBM with volume exclusion, variations of which have been used to investigate cell invasion (Plank and Simpson 2013; Irons et al. 2016), and crowding and adhesion effects (Plank and Simpson 2012; Johnston et al. 2013; Middleton et al. 2014). Alternative to moment closure and mean-field approximations used in the aforementioned models in this paragraph, Bruna et al. (2017) developed a systematic upscaling method based on matched asymptotic expansions for systems with short range repulsive interactions. Applying this method to a system of interacting Brownian particles, they derived a nonlinear diffusion model for biological population density where the nonlinear terms account for various types of interactions.

## Integro-partial differential equation models

It is likely that in many biological processes cell–cell and cell–tissue interactions are not only local, as typically assumed in continuum PDE models, but also have non-local components. For example, during collective migration newt pigment cells can extend their ligand/receptor carrying filopodia ten times an average cell diameter (Tucker and Erickson 1986). Armstrong et al. (2006) and Hillen (2006) were amongst the first to use integro-PDEs to model cell–adhesion and chemotaxis/cell–tissue interactions, respectively. Armstrong et al. (2006) constructed their model by considering the forces acting on cells in a conservative system. Using mass conservation they described the evolution of cell density in one dimension as:

5 $$\begin{aligned} \frac{\partial p}{\partial t} = D\frac{\partial ^2 p }{\partial x^2} - \frac{\partial J_a}{\partial x}, \end{aligned}$$

where *p*(*x*, *t*) is the cell density at position *x* at time *t*, *D* is the diffusion coefficient and $J_a$ is the adhesive flux. The adhesive flux accounts for non-local cell interactions and is modelled using

$$\begin{aligned} J_a = \frac{\phi }{R} p F, \end{aligned}$$

where $\phi$ is a constant of proportionality related to viscosity of the medium, *R* is the cell sensing radius and *F* is the total force acting on cells. They assumed that the forces depended on the cells at distance $x_0 < R$ with respect to the cells at position *x*. Therefore, the total force was of the form

6 $$\begin{aligned} F(x,t) = \int _{-R}^{R} \alpha g(p(x+x_0,t))\omega (x_0)dx_0, \end{aligned}$$

where $\alpha$ is a positive parameter reflecting the strength of adhesive force between cells, $g(p(x + x_0),t)$ describes the nature of the forces and their dependence on the local cell density, and $\omega (x_0)$ describes how the direction and magnitude of the force alters according to $x_0$. In other words, the cell at position *x* experiences adhesive forces exerted by cells closer than a distance *R* from it. The form of this kernel is chosen based on the Chapman–Kolmogorov equation for a stochastic process (Pillai and Papoulis 2002). Armstrong et al. (2006) demonstrated, using analytical and numerical methods, that their model is capable of replicating biological processes that involve cell adhesion, such as aggregation of dissociated cells and the active sorting process of different cell types from a randomly distributed mixture.

Chauviere et al. (2007) used an integro-PDE framework to incorporate cell–cell and cell–extracellular matrix (ECM) interactions. Their model takes into account haptotactic and chemotactic effects, as well as cell–cell and cell–ECM interactions. They used the following transport equation to describe cell movement

7 $$\begin{aligned} \frac{\partial p}{\partial t} + \varvec{v} \cdot \nabla _{\varvec{x}} p + \nabla _{\varvec{v}} \cdot [\varvec{f}(c) p] = J_c + J_m, \end{aligned}$$

where $p(t,\varvec{x},\varvec{v})$ is cell density at time $t\ge 0$ and location $\varvec{x}\in \textit{D} \subseteq {\mathbb {R}}^3$, with velocity $\varvec{v} \in \textit{V} \subseteq {\mathbb {R}}^3$. They assumed a constant initial distribution of cells and zero Dirichlet boundary conditions. The function $\varvec{f}(c) \in {\mathbb {R}}^3$ models the effect of chemotaxis with $c(t,\varvec{x})$ denoting the chemotactic signal, while $J_c$ and $J_m$ describe cell–cell and cell–ECM interactions, respectively. These interaction terms are proposed to take the form (for $J_c$)

8 $$\begin{aligned} J_c(\varvec{v})&= \int _V \int _V \nu _c(\varvec{v'},\varvec{v'}_*) \psi _c ((\varvec{v'},\varvec{v'}_*) \rightarrow \varvec{v}) p(t,\varvec{x},\varvec{v'})p(t,\varvec{x},\varvec{v'}_*) \text {d}\varvec{v'}\text {d}\varvec{v'}_* \nonumber \\&\quad - \int _V \int _V \nu _c(\varvec{v},\varvec{v'}_*) \psi _c ((\varvec{v},\varvec{v'}_*) \rightarrow \varvec{v'}) p(t,\varvec{x},\varvec{v})p(t,\varvec{x},\varvec{v'}_*) \text {d}\varvec{v'}\text {d}\varvec{v'}_*, \end{aligned}$$

where $\nu _c(\varvec{v'},\varvec{v'}_*)$ is the encounter rate, the number of encounters per unit volume and unit time between cell pairs with velocities $\varvec{v'}$ and $\varvec{v'}_*$, and $\psi _c ((\varvec{v'},\varvec{v'}_*) \rightarrow \varvec{v})$ denotes the probability of the transition of a cell having velocity $\varvec{v'}$ before encounter to continue its motion with velocity $\varvec{v}$ after having interacted with a cell having a velocity $\varvec{v'}_*$. Put simply, this represents how the density of cells travelling at speed $\varvec{v}$ changes due to encounters with cells at the same position and time travelling at different speeds. The kernel $J_m$ is defined in a similar manner to incorporate changes resulting from cell encounters with ECM fibres, which are assumed to be remodelled by cell-induced degradation described by a Boltzmann-like interaction term. It is of the form

9 $$\begin{aligned} J_m(\varvec{v})&= \int _V \int _{S^2_+} \nu _m(\varvec{v'},\varvec{n'}) \psi _m ((\varvec{v'},\varvec{n'}) \rightarrow \varvec{v}) p(t,\varvec{x},\varvec{v'})m(t,\varvec{x} ,\varvec{n'}) \text {d}\varvec{v'}\text {d}\varvec{n'} \nonumber \\&\quad - \int _V \int _{S^2_+} \nu _c(\varvec{v},\varvec{n'}) \psi _m ((\varvec{v},\varvec{n'}) \rightarrow \varvec{v'}) p(t,\varvec{x},\varvec{v})m(t,\varvec{x} ,\varvec{n'}) \text {d}\varvec{v'}\text {d}\varvec{n'}, \end{aligned}$$

where $m(t,\varvec{x} ,\varvec{n})$ is the density and orientation of the fibrous ECM, $\nu _m(\varvec{v'},\varvec{n'})$ is the encounter rate of a cell with velocity $\varvec{v'}$ with a fibre with orientation $\varvec{n'} \in S^2_+$ and $\psi _m ((\varvec{v'},\varvec{n'}) \rightarrow \varvec{v})$ denotes the probability of the transition of a cell having velocity $\varvec{v'}$ before the encounter to continue its motion with velocity $\varvec{v}$ after having interacted with a fibre oriented along $\varvec{n'}$. Using this novel approach to include cell–cell and cell–ECM interactions, Chauviere et al. (2007) were able to provide insights into how cells behave in anisotropic and heterogeneous ECM distributions. Further work on the application of integro-PDEs to investigate the importance of cell–cell and cell–ECM adhesion in cancer have been carried out by, for example, Gerisch and Chaplain (2008), Gerisch and Painter (2010) and Chaplain et al. (2011).

A completely different application of integro-PDEs to model collective migration is that of Colombi et al. (2015a, b). They used a measure theoretic approach to develop a modelling framework for heterogeneous cell populations, for example, tip and stalk cells in blood vessel formation and development, in which tip cells guide the rest of the cells. Colombi et al.’s (2015a, b) model is a hybrid model wherein specialised cells (e.g. tip cells) are considered as discrete, and are coupled to unspecialised cells (e.g. stalk cells) modelled using continuum approach. Their approach provides a mathematically rigorous framework to incorporate cell-level concepts, such as cell interaction radii and forces, into the dynamics of populations. Their model is particularly useful for biological systems with multiple cell populations which individually require a distinct type of mathematical description according to their specific functions. There are many mathematical models that use integro-PDEs to account for larger phenotypic variability in cancer (Lorenzi et al. 2016, 2018; Busse et al. 2016), but we do not describe them in detail in this review.

Recently, Buttenschön et al. (2018) have developed a framework within which non-local adhesion and chemotaxis integro-PDE models can be derived from a space-jump process. They verified their results by comparing their continuum model with the mean-field behaviour of the stochastic random walk model for a large total number of cells and constant diffusion and advection–diffusion coefficients. These results demonstrate how the limiting behaviour of certain IBMs can be accurately described by integro-PDEs, which can be used to model non-local interactions with parameters for microscale properties directly inferred from experimental data. Furthermore, this allows us to incorporate assumptions made on behaviour at the microscopic level into the macroscopic description in a systematic fashion.

## Domain growth

Thus far, we have focused on models defined on fixed (non-growing) spatial domains. However, there are many cases where domain growth occurs on the same timescale as the phenomena being modelled and so must be taken into account. One of the most spectacular examples of the effects of domain growth is in the pigmentation patterns on certain fishes, which change qualitatively due to domain growth. It was shown that this is consistent with a Turing reaction–diffusion model solved on a growing domain (Kondo and Asai 1995). In the context of collective cell migration, we define the domain to be the tissue through which, or on which, the cells of interest migrate. Depending on the model type, discrete or continuous, lattice-based or off-lattice, there are different ways to incorporate domain growth. Therefore, we now proceed with a description of the techniques used to model a growing domain.

In continuum reaction–diffusion models, the effect of domain growth is described by an extra advection term, $\nabla \cdot (\varvec{a} p)$, in the reaction–diffusion equation:

10 $$\begin{aligned} \frac{\partial p}{\partial t} + \nabla \cdot (\varvec{a} p) = D\nabla ^2 p + f(p), \end{aligned}$$

where *p* is the density of cells, *D* is the diffusion coefficient, $\varvec{a}$ is the velocity field generated by domain growth: $\varvec{a} \cdot \nabla p$ corresponds to the transport of material around the domain at a rate determined by the flow, $p\nabla \cdot \varvec{a}$ corresponds to the diluting effect of local volume increase, and *f*(*p*) represents a reaction function. Crampin et al. (1999, 2002) provided a detailed derivation of the above reaction–diffusion Eq. (10) for spatially uniform domain growth, and a few examples of non-uniform in space and time domain growth. Crampin et al. (2002) demonstrated how the sequences of patterns change for different domain growth profiles, giving a further mechanism for controlling pattern selection. Landman et al. (2003) demonstrated that the same ideas can be extended to incorporate uniform domain growth into continuum models for cell migration that include chemotaxis. Simpson et al. (2006) built on their work and provided an efficient numerical algorithm for solving PDE models of chemotactic and diffusive migration on a non-uniformly growing domain. In addition, they provided rigorous mathematical results that show how different types of domain growth impact cell invasion.

In the continuum models on a growing domain described above, the PDEs were solved numerically. However, Simpson (2015) derived an exact solution to PDE models of linear reaction–diffusion processes on a uniformly growing domain. They used their analytical solution to further investigate the interplay between different parameters and model features, such as the rate at which the domain elongates and the diffusion coefficient. This is a good example of how a mathematically tractable model can be used to provide insights to the biology.

It is important to remark that it is challenging to implement integro-PDE models on a growing domain due to changes in the domain of integration of the interaction terms $J_c$ and $J_m$ in Eq. (7). To our knowledge there are no studies in this area, in particular, when combined with cell migration.

Baker et al. (2010) demonstrated how mathematical models that include domain growth at a microscopic level, referred to here as a discrete description, translate to the macroscopic description, referred to here as a continuum description. They used a one-dimensional lattice-based IBM to demonstrate the correspondence between microscopic and macroscopic modelling frameworks. In their model cells were guided by external signals, for example, those due to the presence of a chemottractant. They incorporated domain growth by the instantaneous doubling in size and division of underlying lattice elements. They obtained good correspondence between the microscopic and macroscopic frameworks for slow growth (smaller than quadratic). Yates et al. (2012) extended these studies and demonstrated that different ways to split the lattice sites ensures that individual-based stochastic simulations provide equivalent results to PDE models on any non-uniformly growing domain.

Ross et al. (2016, 2017) investigated different ways to implement domain growth in lattice-based models. They simulated a one-dimensional random walk with volume exclusion, cell proliferation and death. They demonstrated that changes in the way new sites are added to the lattice influence steady-state densities and spatial correlations. Their analyses provided evidence that to model domain growth accurately, detailed experimental data on the underlying tissue growth must be incorporated to avoid inaccurate predictions from a model.

While most of the work described in this section is theoretical, the authors discuss how their suggested frameworks could be applied to model biological processes such as the migration of melanoblasts through the developing dorsal lateral epithelium in the embryonic mouse (Wilkie et al. 2002) and morphogenesis in general (Aman and Piotrowski 2010). An application of an IBM on a growing domain to investigate the formation of diffuse clones, chimeric stripes and belly spots induced by migration of melanoblasts in mouse chimeras has been carried out by Mort et al. (2016). They demonstrated that melanoblast colonisation is likely to proceed through a process of undirected migration, proliferation and tissue expansion, and that reduced proliferation is the main cause of the belly spots in mouse.

## Neural crest cell migration

We focus now on the NC illustrating that it is a diverse and very tractable experimental collective cell migration model system. NC cells are unique to vertebrates and their migration is fundamental in embryonic development. NC cells are derived from the ectoderm at the crest of the neural tube, a structure which forms the spinal cord and brain, and are released to migrate away from the neural tube to colonise different sites. There are distinct NC cell populations that emerge at different axial levels of the dorsal neural tube and migrate in streams which appear to be confined to specific corridors of the tissue domain (Fig. 1). Cranial/cephalic NC cells contribute to mainly cartilage, bone, teeth, connective tissue and cranial neurons. Cardiac NC cells contribute to cartilage and connective tissue of the cardiovascular system. Trunk NC cells give rise to melanocytes, glia and neurons of the peripheral nervous systems. Vagal/enteric and sacral NC cells give rise to the ganglia of the enteric nervous system (Le Douarin 2004; Rogers et al. 2012). If NC cells fail to reach a target, or populate an incorrect location, then improper cell differentiation or uncontrolled cell proliferation may result, which can further lead to developmental defects and diseases, for example Hirschsprung’s disease, characterised by a failure of NC cells to fully migrate throughout the developing gut (Landman et al. 2007).

In NC, many mathematical models have been developed to address different collective NC cell behaviours observed within and across species. We summarise here a few of the key studies, and refer the reader to some reviews on the wide range of literature on NC cell migration (Kulesa et al. 2010; Schumacher et al. 2016; Szabó and Mayor 2016, 2018).

### Individual-based models

As discussed in Sect. 2, an advantage of IBMs over continuum models is that they can incorporate individual-cell-level properties more easily. Therefore, they are the most commonly used models to simulate the migration of NC cells. We provide examples of these models in the following two subsections: on a fixed domain and on a growing domain.

#### Fixed domain

An agent-based model on a two-dimensional fixed lattice was used to model collective cell motility of cephalic NC cells in *Xenopus* driven by contact inhibition of locomotion (CiL) and co-attraction (CoA) (Carmona-Fontaine et al. 2011). CiL is a process during which migratory cells momentarily stop upon physical contact and subsequently repolarise to move in the opposite direction, whereas CoA describes a mutual cell–cell attraction. The importance of CiL for the migration of NC cells was demonstrated using *Xenopus* and zebrafish cephalic NC cells (Carmona-Fontaine et al. 2008; Theveneau et al. 2010; Mayor and Carmona-Fontaine 2010). Further evidence for CiL has been recently provided by Roycroft et al. (2018), who showed how CiL could arise via the redistribution of adhesive forces through Src/FAK (steroid receptor coactivator and focal adhesion kinase). The existence and necessity of the opposite phenomenon, namely CoA, to ensure that the cells remain in a group, has been verified in *Xenopus* cephalic NC cells (Carmona-Fontaine et al. 2011).

In their IBM Carmona-Fontaine et al. (2011) incorporated short-range repulsive interactions aimed to emulate CiL, and longer-range attractive interactions for CoA. CiL was modelled by enforcing alignment of cells after collisions and then inducing a repulsive force with some noise. CoA was modelled in two ways: by enforcing cells within a certain radius to move towards each other, or based on diffusion of a co-attractant. Both implementations produced similar results. Their modelling results supported conclusions drawn from experimental findings on these guidance mechanisms, i.e. that the combination of CiL and CoA is essential for cells to self-organise and respond efficiently to external signals. Woods et al. (2014) further extended Carmona-Fontaine et al.’s model and incorporated elastic collisions to account for the deformation of cell shape. Their simulation results reinforced the findings that CiL and CoA are together sufficient mechanisms for successful invasion in cephalic NC cells in *Xenopus*.

Another model which includes the biological assumptions of CiL and CoA was developed by Szabó and Mayor (2016). They developed a Cellular Potts model to investigate the effect of confinement on the collective migration of NC cells. The optimal number of cells in a given confinement width predicted by their computational results coincided with the width of NC migratory streams across different species. They suggest that this may explain the evolutionarily conserved nature of NC streams.

The studies described in this section provide explanations for certain important phenomena in collective cell migration, such as cell–cell interactions and domain confinement. Nevertheless, there is evidence that for other types of NC cells external guidance cues play an important role in collective NC cell migration. For example, vascular endothelial growth factor (VEGF) for chick cranial NC cells and stromal cell-derived factor (SDF1) for *Xenopus* cranial NC cells have been shown to act as chemoattractants (McLennan et al. 2010; Theveneau et al. 2010; Kasemeier-Kulesa et al. 2010). Therefore, while the modelling studies described in this section have elucidated a potential mechanistic basis for collective migration, it is important to note that the variety of behaviours observed in NC migration suggest that there are other mechanisms that can give rise to collective cell migration.

#### Growing domain

The above models were all implemented on fixed (non-growing) domains. We now proceed to review some models for different NC cell systems that incorporate domain growth and external cell guidance cues.

The computational and experimental studies by McLennan et al. have provided a number of insights into the migration of chick cranial NC cells (McLennan et al. 2012, 2015a, b, 2017). We provide a more detailed description of this model, reproduce their results and suggest future directions based on their work. Their key results included evidence of guidance via a cell-induced gradient of VEGF, which acts as a chemoattractant, heterogeneity of cell phenotypes, namely “leaders” and “followers”, and possibility of phenotypic transition. To model cell dynamics they used a two-dimensional off-lattice IBM with volume exclusion, which was coupled to a continuum reaction–diffusion model for the dynamics of the chemoattractant VEGF. The cell positions were updated in the following way: leaders undertake a biased random walk up a cell-induced gradient of chemoattractant, whereas followers are guided by the leaders, if sufficiently close to them, or move randomly. The exact guidance mechanism is unknown, therefore McLennan et al. (2012, 2015a, b, 2017) made the assumption that leaders guide followers by physical contact via filopodia, giving rise to chain-like structures, which are observed experimentally (Wynn et al. 2013). They included a phenotype switching mechanism between the cell phenotypes; in the absence of VEGF, lead cells acquire follower cell behaviour. The dynamics were modelled on a rectangular growing domain in the x direction (from the neural tube to the target tissue). They assumed that the growth was spatially uniform but non-uniform in time. Initially, the domain is empty of cells, which then enter at a constant rate at the left-hand boundary, provided that there is space available. The cells satisfy zero flux boundary conditions on the other three boundaries. McLennan et al. used Eq. (11) as a (non-mechanistic) description of domain growth in the *x* direction with a sufficient number of parameters to fit experimental data:

11 $$\begin{aligned} L(t) = L_0 \left( \frac{L_\infty e^{a(t-t_s)L_\infty }}{L_\infty - 1 + e^{a(t-t_s)L_\infty }} +1 - \frac{L_\infty e^{a(-t_s)L_\infty }}{L_\infty - 1 + e^{a(-t_s)L_\infty }} \right) , \end{aligned}$$

with $a ,t_s,L_{\infty },L_0 >0$ parameters inferred from experimental results and *L*(*t*) is the domain length (McLennan et al. 2012). They incorporated uniform domain growth in the dynamics of cells by updating the positions of cells at each timestep. The VEGF dynamics were modelled as follows:

12 $$\begin{aligned} \frac{\partial c}{\partial t}= & {} \underbrace{D \left( \frac{1}{L^2} \frac{\partial ^2 c}{\partial x^2}+ \frac{\partial ^2 c}{\partial y^2}\right) }_{(1)} -\underbrace{c \sum _{i=1}^{N(t)}\frac{\lambda }{2\pi R^2} \text {exp}\left[ -\frac{L^2(x-x_i)^2 + (y-y_i)^2}{2R^2} \right] }_{(2)} \nonumber \\&+ \underbrace{\kappa c(1-c)}_{(3)}- \underbrace{\frac{\text {d}L}{\text {d}t} \frac{1}{L}c}_{(4)}, \end{aligned}$$

where *c*(*x*, *y*, *t*) represents the concentration of chemoattractant at position $$ x \in [0,L(t)]$$, $$y\in [0,L_y]$$ (for $$L_y>0$$ constant) and time *t*. The terms on the right-hand side of Eq. (12) correspond to: (1) diffusion of chemoattractant with constant diffusion coefficient *D*, with the $$1/L^2$$ factor due to the domain growth in the *x* direction (*L*(*t*) is the length of the domain); (2) internalisation of chemoattractant by cells; (3) production of chemoattractant; (4) dilution of chemoattractant due to domain growth. The other parameters are as follows: *R* is the cell radius, $$\lambda $$ is the internalisation rate, $$\kappa $$ is the production rate of chemoattractant and $$(x_i,y_i),$$$$i=1,\dots ,N(t)$$, is the position of the centre of cell *i*, where *N*(*t*) is the number of cells at time *t*. They assumed zero flux boundary conditions and initial conditions $$c(x,y,0) \equiv 1$$. We refer the reader to the supplementary information of McLennan et al. (2015a, b, 2017) for full details of the model.

**Fig. 2:**
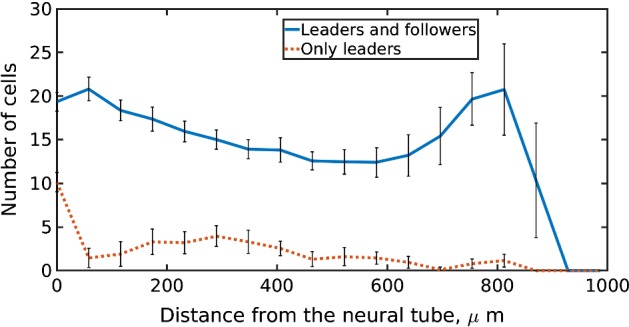
Simulation of the McLennan et al. (2015a, b, 2017) model (see text for details). Distribution of cells along the domain when there are two different cell phenotypes, namely leaders and followers, and when all the cells are leaders, average of 20 simulations after 24 h. Error bars represent standard deviation

**Fig. 3:**
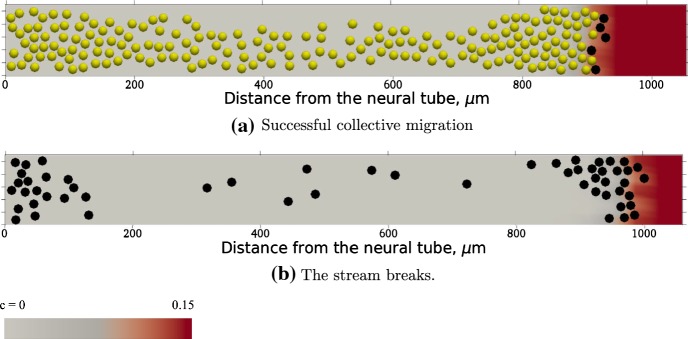
Snapshots at $t = 24$ h from simulations of the model developed based on McLennan et al.’s (2015a, b, 2017) model (see text for details). Black circles correspond to leader cells, yellow circles correspond to follower cells, and *c* is the concentration of chemoattractant. In the case of two cell phenotypes (**a**), in which leaders guide followers by forming contact “chains”, the cells migrate collectively. If there is only cell type with cells that are guided by a cell-induced VEGF gradient, then the stream breaks (**b**) (color figure online)

McLennan et al.’s (2012) study shows that it is possible to generate coherent cell migration with two phenotypes. We reproduce this result by showing that successful invasion is not possible when there is only one cell phenotype, but the invasion is robust when there are two cell phenotypes (Figs. 2, 3). However, there may well be other phenotypes expressing different movement properties, so there may be more than two cell phenotypes. In fact, recent computational studies have demonstrated that cell states do not necessarily have to be discrete and a continuum transition between cell states offers a functionally equivalent description (Schumacher 2019). Measurements of the whole transcriptome support the hypothesis of a continuum of cell states.

In the above, McLennan et al. (2012, 2015a, b, 2017) have assumed, for simplicity, that domain growth is uniform in space and non-uniform in time. This assumption was based on simple length measurements of the entire NC migratory domain throughout successive developmental stages. However, recent experiments that measure cell proliferation and cell density changes of the mesoderm through which NC cells travel suggest that this is not the case, and we are presently investigating the effects of this on model predictions (results not shown).

Another IBM, which also incorporates domain growth, was used to model development of the embryonic gut of avian embryos (Binder et al. 2012; Cheeseman et al. 2014; Newgreen et al. 2009; Zhang et al. 2010). In this study, domain growth was incorporated using random insertion of lattice sites. Different types of domain growth can be implemented in this framework, including spatially non-uniform growth. However, the effect of spatially non-uniform domain growth on collective cell migration and successful colonisation of the domain in hybrid or continuum models, for example the McLennan et al. (2012, 2015a, b, 2017) model with continuum reaction–diffusion for chemoattractant, are still open questions.

### Continuum models

Continuum modelling has been used to study the migration of NC cells to provide biological insights. For illustrative purposes, we provide an example of a particular PDE model and an integro-PDE model for different types of NC cell migration. We identify their main assumptions and key results.

#### Partial differential equation model

Simpson et al. (2007) used a continuum model to investigate the roles of proliferation and migration for successful invasion and colonisation of the gut by vagal NC cells that establish the enteric nervous system. More specifically, they were looking for the main causes that inhibit NC cells from reaching the end of the gut, which can result in abnormal development (Newgreen et al. 1996). They developed their model based on experimental studies of chick and quail embryos. Simpson et al. (2007) proposed a coupled system of PDEs for the two cell types, which were observed to differ experimentally, and described migration with diffusion, and proliferation with logistic growth:

13 $$\begin{aligned} \frac{\partial D}{\partial t}= & {} \alpha _D \frac{\partial ^2 D}{\partial x^2} + \lambda _D D \left[ 1 - \left( \frac{D+H}{C} \right) \right] , \end{aligned}$$

14 $$\begin{aligned} \frac{\partial H}{\partial t}= & {} \alpha _H \frac{\partial ^2 H}{\partial x^2} + \lambda _H H \left[ 1 - \left( \frac{D+H}{C} \right) \right] , \end{aligned}$$

where $x\in [0,L]$, for $L\ge 0$ constant and $t \ge 0$. *D*(*x*, *t*) denotes donor cell density (usually from quail), *H*(*x*, *t*) host cell density (usually from chick), *C* is the carrying capacity, $\alpha _D,\alpha _H \ge 0$ are the respective motility rates and $\lambda _D, \lambda _H \ge 0$ are the respective proliferation rates. They considered the cases in which donor and host cells were identical and the case in which donor cells were non-proliferative, i.e. $\lambda _D = 0$. They used different initial conditions for donor cells, *D*(*x*, 0), relative to the host cells, *H*(*x*, 0), to qualitatively replicate the initial state of invasion in graft experiments. Their results are robust to boundary conditions, that is, they could be set either to no flux or appropriate Dirichlet conditions.

Simpson et al. (2007) analysed their model mathematically and provided predictions that were validated experimentally. In particular, they identified that cell invasion waves are organised in such a way that the NC cells at the front of the stream are responsible for proliferation and motility, while the NC cells behind them are not proliferative and do not contribute to the invasion of unoccupied tissues. These differences were not induced by intrinsic cell properties (verified using their mathematical model and grafting experiments involving identical and non-identical cells) but whether or not the cells were adjacent to NC-free tissue. Their modelling revealed the importance of proliferation to carrying capacity in the system. The model was further used to investigate the reasons for developmental defects, including Hirschsprung’s disease (Landman et al. 2007). Hirschsprung’s disease is a motor disorder of the gut, which is caused by the failure of NCs to fully invade and populate the gut. Landman et al.’s (2007) results revealed that directional invasion of the gut is driven by a combination of NC cell proliferation and migration. This suggests that the experimental focus should be to examine the mechanisms that regulate the balance between cell proliferation and migration.

#### Integro-partial differential equation model

Another continuum approach to model NC cells was considered by Painter et al. (2015). They investigated different NC cell–cell interactions through a non-local PDE modelling framework. This is a potentially promising approach because there is no evidence that such interactions are purely local, for example, cranial and trunk NC cells often use long membrane protrusions, such as lamellipodia and filopodia, to sense their non-local extracellular environment (Clay and Halloran 2010; Painter et al. 2015). Painter et al. (2015) used an abstracted phenomenological argument, based on a classical continuum approach (Murray 2002), to include mechanisms such as CiL and CoA between NC cells (described in Sect. 6.1.1). We provide a short description of their model to convey the main idea of their approach. Painter et al. (2015) developed a modelling framework for an *n*-dimensional system, but restricted attention to a one-dimensional case for their application to model NC cells. They considered a mass conservation equation for cell density, $p(\varvec{x},t)$:

15 $$\begin{aligned} \frac{\partial p }{\partial t} = - \nabla \cdot \varvec{J} , \end{aligned}$$

where $\varvec{J}$ represents the flux and $\varvec{x} \in {\mathbb {R}}^n$. The flux consisted of two parts, namely random, and interaction, $\varvec{J} = \varvec{J}_{random} + \varvec{J}_{interaction}$. The random flux was defined as $\varvec{J}_{random} = -D_{ p} \nabla p$, where $D_{ p}$ is the constant cell diffusion coefficient. The interaction flux was of the form

16 $$\begin{aligned} \varvec{J}_{interaction}(\varvec{x},t) = p f( p) \omega \int _{{\mathbb {R}}^n }\frac{\varvec{s}}{|\varvec{s}|} \Omega (|\varvec{s}|;\xi ,\mu )g( p(\varvec{x}+\varvec{s},t))\text {d}\varvec{s}, \end{aligned}$$

where $\omega$ is a proportionality constant that depends on factors such as viscosity of the medium, *f*(*p*) is a packing or volume-filling function, $\Omega (|\varvec{s}|,\xi ,\mu ) : [0,\infty ) \rightarrow \mathbb {R}$ is the interaction function, $\xi$ is the interaction range, $\mu$ is the interaction strength, and $g( p(\varvec{x}+\varvec{s},t))$ is the functional dependence on cells at $\varvec{x}+\varvec{s}$, which corresponds to the strength of the force exerted on cells at $\varvec{x}$ due to the cell density at $\varvec{x} +\varvec{s}$. They used different forms of the interaction function, such as a step function or exponential function, to incorporate CiL and CoA.

Painter et al. (2015) also extended this model to include two populations with varying interactions, but used a one population model to investigate NC cell migration and provided simulation results that demonstrate how interaction strength and range affect the invasion depth and dispersal. They observed that the solutions appear to evolve to travelling wave profiles. However, it is still an open question to determine the dependence of the wave speed on the parameters $\mu$ and $\xi$ analytically. Painter et al. (2015) hypothesised that if NC cells communicated at longer distances than previously assumed by Carmona-Fontaine et al. (2008), then the invasion could be significantly enhanced. These results suggest that it could be fruitful experimentally to look for mechanical or chemical interactions between cells at different distances via, for example, an extension of filopodia of varying length.

## Discussion

In this review, we provided a brief summary of a range of mathematical modelling frameworks (IBM, PDE, integro-PDE) for collective cell migration and discussed their relative strengths and weaknesses. We considered examples of how continuum models can be derived from IBMs. We discussed integro-PDEs and their usefulness for incorporation of non-local cell–cell and cell–tissue interactions. We identified that domain growth can have an important impact on collective cell invasion and provided a short summary of techniques used to model growth in both discrete and continuum frameworks. We pointed out how continuum models can be derived, on fixed or growing domain, from IBMs allowing us to directly relate population level properties to individual cell-level properties.

We considered the paradigm case of the highly migratory, embryonic NC to demonstrate how different modelling frameworks can be used to answer biological questions. Interdisciplinary studies of NC cell migration have revealed a huge variety of cell–cell and cell–tissue interactions across various NC cell types and different species. Mathematical modelling has helped us to understand some of these behaviours and to identify further open questions. There are still many other cell–cell/cell–tissue interactions that may contribute to cell invasion. Furthermore, variations in the tissue, such as tissue growth or chemical composition, are also likely to have an impact on collective cell migration. Recent advances in imaging technology and single cell analyses now mean that, experimentally, we can track individual cell behaviours and interrogate gene expression changes to generate multiscale spatiotemporal data. One of the ongoing challenges in this field is to incorporate spatial statistics into modelling frameworks for parameter identification and model refinement (Warne et al. 2018). This will allow more precise model predictions and validation.
